# Healthcare-associated outbreak of severe acute respiratory coronavirus virus 2 (SARS-CoV-2) in a rural hospital in Missouri, March 2020

**DOI:** 10.1017/ice.2020.1281

**Published:** 2020-10-20

**Authors:** George Turabelidze, Betty C. Faulconer, Steven J. Lawrence, Amy Pierce, Brittany K. Smith, Daved H. Fremont

**Affiliations:** 1 Missouri Department of Health and Senior Services, Jefferson City, Missouri; 2 Washington University School of Medicine, St Louis, Missouri

*To the Editor—*Prevalence of SARS-CoV-2 infection is as high or higher among HCP than the general population.^1–3^ In a prospective, observational cohort study in the United Kingdom and the United States, frontline healthcare workers had at least a 3-fold increased risk of reporting a positive COVID-19 test and predicted COVID-19 infection, compared with the general community.^4^ A recent living review of multiple studies of coronavirus infections found increased burden in HCP, and the infection incidence was higher after unprotected exposures.^5^ The prevalence of SARS-CoV-2 antibodies was lower among personnel who reported always wearing a face covering while caring for patients, compared with those who did not.^6^ Despite accumulating data, the full potential of SARS-CoV-2 transmission in a hospital setting is not fully understood.

On March 11, 2020, a 77-year-old man hospitalized in Missouri was diagnosed with coronavirus disease 2019 (COVID-19) after testing positive for severe acute respiratory coronavirus virus 2 (SARS-CoV-2) by a real-time reverse-transcriptase polymerase chain reaction (real-time RT-PCR) test. During the 2 ER visits and a 3-day hospitalization, 60 healthcare personnel (HCP) at hospital A were exposed to this patient while not using appropriate personal protective equipment (PPE). We conducted a public health investigation to determine the scope of SARS-CoV-2 transmission to HCP during the unprotected exposure to the patient with COVID-19.

Nasopharyngeal (NP) specimens collected from all HCP who developed symptoms of possible COVID-19 during their 14-day quarantine were tested for SARS-CoV-2 using real-time RT-PCR at the Missouri State Public Health Laboratory. Serological testing of blood specimens from symptomatic and asymptomatic HCP was conducted using a research laboratory-developed IgG antibody-capture ELISA. Recombinant nucleocapsid and trimeric spike antigens were cloned from the 2019-nCoV/USA-WA1/2020 strain and expressed in *E.coli* and Expi293F cells, respectively. Heat-inactivated sera were scanned at a 1:500 dilution against SARS-CoV-2 trimeric spike and nucleocapsid proteins along with influenza hemagglutinin (positive control) and bovine serum albumin (negative control). Samples were considered COVID-19 positive if they had an optical density >1 against both SARS-CoV-2 antigens.

Of the exposed HCP, 34 (57%) developed respiratory and/or constitutional symptoms within a median of 3 days (range, 0–7) after exposure. All 34 symptomatic HCP were tested for SARS-CoV-2 by PCR at a median of 3 days (range, 1–9) from symptom onset. One HCW (2.9%) with multiple unprotected exposures to the index case in the ICU was positive for SARS-CoV-2. Of the 60 exposed HCP, 27 (45%) were tested for SARS-CoV-2 antibodies at a median of 41 days (range, 37–46) after last exposure to the index patient (Table 1). Of those, 19 (70%) reported COVID-19–compatible illness during the monitoring period. Of 27 exposed HCP who previously tested negative by PCR on day 6 and day 7 postexposure to index case, 2 (7.4%) tested positive by ELISA. Both were nurses and became ill 4 and 5 days, respectively, following exposure to the index case. The serological positivity among symptomatic HCP was 2 of 19 (10.5%). Overall positivity among those who had both PCR and ELISA tests performed was 2 of 17 (11.8%). Both HCP with SARS-CoV-2 antibodies had a high-risk exposure during several close contacts with the index patient. One nurse had a total duration of exposure up to 60 minutes during the patient’s second emergency department visit on day 8 of illness. The other nurse had a total duration of exposure up to 30 minutes on the medical ward during day 2 of hospitalization on day 9 of the case patient’s illness. Both had only medical gloves on, but neither was using a gown, face mask, or eye protection. Both nurses had been coughed upon while <2 m (6 feet) from the index patient without source control in place.

**Table 1.** Characteristics of Healthcare Personnel Tested for SARS-CoV-2 Antibodies

Characteristic	No. (%) (N=27)
Age, median y (range)	34 (18–67)
Sex	
Female	22 (81)
Male	5 (19)
Symptoms of possible COVID-19	
Yes	19 (70)
No	8 (30)
CDC exposure risk category	
High	25 (93)
Medium	2 (7)
Low	0
No risk	0
Hospital positions	
Nurse	6 (22)
Radiology technician	5 (19)
Respiratory therapist	5 (19)
Nursing assistant	3 (11)
EMT	3 (11)
Lab	2 (7)
Physician	1 (4)
Registration	1 (4)
Physical therapist	1 (4)
Blood sample collection days after exposure, median (range)	41 (37–46)

Note. CDC, Centers for Disease Control and Prevention; EMT, emergency medical technician.

In our study, only 1 of 34 exposed, symptomatic HCP tested positive for SARS-CoV-2 by PCR, which is comparable to the finding of only 3 of 43 HCP testing positive for SARS-CoV-2 in a small California hospital despite multiple unprotected exposures.^7^ We recognized reasonable probability of false-negative results due to less-than-perfect PCR test sensitivity, and we also considered accumulating data on SARS-CoV-2 shedding patterns, as well as PCR test performance, which depends on the stage of the person’s infection.^8,9^ Therefore, we conducted serological testing of HCP 6 weeks after their last exposure to the index case, and we detected 2 more medical staff with evidence of SARS-CoV-2 infection. Both of them tested negative by PCR during the symptomatic period. One of them experienced severe illness requiring hospitalization, and the SARS-CoV-2 RT-PCR was negative 3 times.

Our findings may indicate a need for both PCR and serological testing modalities during SARS-CoV-2 hospital outbreak investigation. Serological testing of all HCP with unprotected exposure may provide a more comprehensive assessment of the hospital outbreak and may also reveal additional transmission chains that would not otherwise be identified with PCR-only testing.

Our study has several limitations. First, not all exposed HCP participated in the study; therefore, the true number of all transmission events could have been higher. Second, those HCP with antibody titers against the SARS-CoV-2 could have acquired infection outside the hospital setting, even though the timing of their clinical presentation and the lack of widespread community activity of the virus at that time makes this possibility unlikely.

We believe that early recognition and isolation of patients with suspected COVID-19, as well as strong enforcement of proper PPE use, is crucial for the protection of the hospital workforce.
